# Synthesis of a novel coumarin via the Mannich reaction: *in vitro* and *in silico* evaluation of anti-cancer, antimicrobial and antioxidant activities

**DOI:** 10.1098/rsos.250854

**Published:** 2025-10-15

**Authors:** Tran Nguyen Ngoc Huyen, Uyen Nguyen Ngoc Phuong, Tran Nguyen Minh An, Anh Nguyen Thi Hong

**Affiliations:** ^1^ Industrial University of Ho Chi Minh City, Ho Chi Minh City, Vietnam; ^2^ Parkdale Secondary College, Mordialloc, Australia; ^3^ Ho Chi Minh City University of Industry and Trade, Ho Chi Minh City, Vietnam

**Keywords:** coumarin, Mannich, cytotoxicity, antimicrobial activity, antioxidant, *in silico* molecular docking model

## Abstract

The novel coumarin derivatives (**2a**–**2j**) were synthesized via the Mannich reaction and evaluated for anti-cancer, antimicrobial and antioxidant activities. Compound (**2d**) exhibited the most potent cytotoxicity against MCF-7 breast cancer cells (IC_50_ = 2.54 ± 0.12 µM), surpassing camptothecin. Compound (**2b**) showed strong activity against HeLa cervical cancer cells (IC_50_ = 5.23 ± 0.12 µM), while compound (**2a**) demonstrated notable effects on HepG2 liver cancer cells (IC_50_ = 8.57 ± 0.42 µM). All three compounds displayed low toxicity toward normal LLCPK1 kidney cells, with IC_50_ values exceeding 94 µM. Antimicrobial assays revealed that compounds (**2a**) and (**2f**) effectively inhibited *Bacillus subtilis*, with minimum inhibitory concentration values of 25 and 75 µM, respectively. Compound (**2i**) showed the strongest antioxidant effect (SC_50_ = 7.36 ± 0.18 µM), comparable to Trolox (SC_50_ = 6.12 ± 0.15 µM). Molecular docking indicated that compound (**2d**) (pose 232) binds tightly to the 1T8I enzyme (Δ*G* = –9.92 kcal mol^−1^, K_i_ = 0.01 µM), forming key interactions with Arg 488 and Lys 532. Molecular dynamics simulations confirmed the complex’s stability in aqueous solution over 90 ns.

## 1. Introduction

Coumarin (2*H*-1-benzopyran-2-one) is a naturally occurring aromatic organic compound, first isolated in 1820. It was initially extracted from tonka beans (*Dipteryx odorata*) and later identified as a distinctive substance. The name ‘coumarin’ is derived from ‘coumarou’, the French term for the tonka bean tree, reflecting its botanical origin [1]. This compound is widely distributed across various plant families, including Fabaceae, Apiaceae and Rutaceae, where it is biosynthesized through the shikimic acid pathway, with phenylalanine serving as a precursor [2]. Coumarin and its derivatives represent a significant class of compounds in natural product research, synthetic chemistry and pharmacological applications due to their diverse biological activities and structural versatility [3]. Their relevance in modern medicinal chemistry has recently been reinforced by Koley *et al.*, who provided a comprehensive review highlighting coumarin-based scaffolds as promising candidates in anti-cancer, antimicrobial and antioxidant drug discovery [4].

Coumarins are a large class of naturally occurring polyphenolic phytochemicals widely distributed in the plant kingdom. The isolation from natural sources, biological or chemical synthesis and pharmacological and biological evaluation of their applications has attracted many organic and medicinal chemists [5]. Typical biological activities such as anti-cancer [6,7], classification of anti-cancer cell mechanisms and design of new drugs with anti-cancer cell activity based on coumarin derivatives are effective strategies [8]. Some coumarin derivatives with heterocyclic rings have high anti-cancer activity [9]. In addition, coumarin derivatives exhibit various other activities, including antioxidant [10,11], antifungal [12], anti-inflammatory [13], antidiabetic [14], anti-HIV [15,16] and anti-SARS-CoV-2 activities [17]. The synthesis of coumarin derivatives has been extensively explored through various methodologies that enhance their structural diversity and biological activity. Various strategies have been employed for the synthesis of phthalhydrazide-coumarin derivatives [14,18], including Pechmann and Knoevenagel condensation under solvent-free conditions [19,20], synthesis under conventional conditions of coumarins [21] and coumarin derivatives carrying imidazole groups [22]. Other approaches include the use of ionic liquid [23], TiCl_4_ catalyst, solvent-free techniques and microwave-assisted synthesis [24]. Among these synthetic strategies, the Mannich reaction has gained particular attention due to its efficiency as a multicomponent reaction for forming carbon–nitrogen bonds, which are critical for enhancing the biological activity of coumarins. This versatility in synthetic approaches not only facilitates the exploration of new coumarin derivatives but also enables the systematic evaluation of structure–activity relationships, providing valuable insights into their pharmacological potential [25].

Currently, the use of computer tools to predict structure–activity relationships is an effective research trend [26]. Various biological simulation modelling methods are employed using software packages such as AutoDock, AutoDock Vina, Surflex-Dock, GOLD (ASP), GOLD (CP), GOLD (CS), GOLD (GS), Hex, ZDOCK, PatchDock and pepATTRACT [27]. Among those solutions, AutoDock software is an effective tool in drug design, easy to implement, with low computer configuration requirements and capable of fast calculation of the number of models, which can range from several hundred to thousands, performed in a few hours [28–30]. The AutoDock tools software package is based on the principle of an experimental free energy function and molecular force field method to evaluate the interaction between a ligand and a macromolecule (receptor: enzyme or protein) through the binding energy value and the ligand interaction model [31]. In this calculation, the determination of the active site on the macromolecule (enzyme/protein) is very important [32]. The value of this model is evaluated through the root-mean-square deviation (RMSD) value [33–35]. If the RMSD value between a reference ligand (ligand existing in the macromolecule) and the ligand of interest is much larger than 2 Å, this model needs to evaluate the stability of the ligand–protein interaction in a real environment using the Gromacs software solution [36–38]. However, no software is completely efficient for a computational model of ligand–receptor interactions [39]. *In silico* absorption, distribution, metabolism, excretion and toxicity (ADMET) modelling is a tool for rational drug design [40]. This modelling is performed through ADMETlab 2.0, an interactive online solution for comprehensive prediction and prediction strategy of ADMET modelling [41]. Among the synthesized compounds in this study, (**2a**) and (**2b**) were previously reported in an early structural study, but without full spectroscopic data or biological evaluation [42]. In this work, these compounds are revisited with a focus on their biological potential alongside other newly synthesized derivatives.

## 2. Material and methods

### 2.1. Materials

#### 2.1.1. Chemical and apparatus

**Scheme 1.**
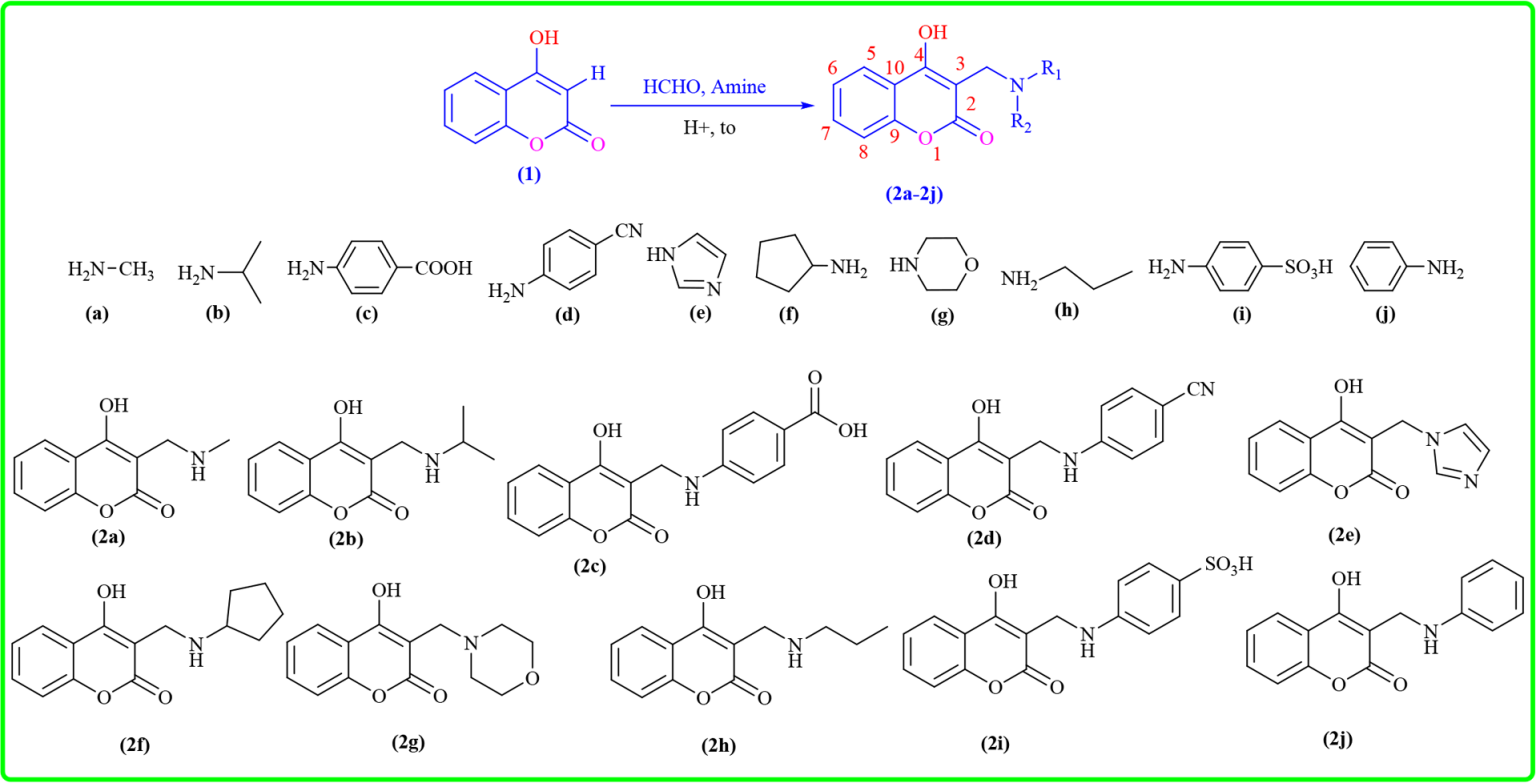
Synthesis of new amine derivatives from 4-hydroxyl coumarin via Mannich reaction.

All reagents were obtained from Aldrich Chemical Company and used as supplied. The (**2a**–**2j**) compounds were synthesized as outlined in Scheme 1. The infrared spectrum was obtained using a Bruker FT-IR Tensor 27 with KBr discs. The nuclear magnetic resonance (NMR) spectra were recorded on a Bruker AM500 for ^1^H- and ^13^C-NMR, using tetramethylsilane (TMS) as the internal standard. A high-resolution mass spectrum (HR-MS) was obtained by electrospray ionization mass spectrometry (ESI-MS) using a Bruker Daltonics micrOTOF-Q II ESI-Qq-TOF spectrometer.

#### 2.1.2. Cell lines and cell culture

Human lung cancer cell line NCI-H460 (HTB-177), human breast cancer cell line MCF-7 (HTB-2), human cervical cancer cells HeLa (CCL-2), human liver cancer cell line HepG2 (HB-8065) and normal kidney epithelial cell line (LLCPK1) were purchased from the American Type Culture Collection (Manassas, Rockville). Cells were cultured at 37°C and 5% CO_2_ in Eagle’s minimal essential medium supplemented with 10% (v/v) FBS (Sigma), 2 mM L-glutamine (Sigma), 20 mM HEPES (Sigma), 0.025 μg ml^−1^ amphotericin B (Sigma), 100 IU ml^−1^ penicillin G (Sigma) and 100 μg ml^−1^ streptomycin (Sigma). Cells used in this study were between passages 4 and 20.

#### 2.1.3. Bacterial strains and culture conditions

The three bacteria*—Escherichia coli (E. coli), methicillin-resistant Staphylococcus aureus (MRSA)* and *Bacillus subtilis (B. subtilis),* and two fungi: *Candida albicans (C. albicans)* and *Trichophyton mentagrophytes (T. mentagrophytes*) were used in this study. The clinical isolates were obtained in 2019 from the Faculty of Biology and Biotechnology, University of Science, Vietnam National University.

### 2.2. Methods

#### 2.2.1. Procedure of the synthesis of coumarin derivatives (**2a**–**2j**)

A mixture of one equivalent of a primary amine derivative or two equivalents of a secondary amine (**2a**–**2j**), 1.8 equivalents of 37% aqueous formaldehyde solution and 0.5 ml of 35% hydrochloric acid was added to a 50 ml round-bottom flask equipped with a reflux condenser and stirred magnetically at 40°C. The reaction mixture initially formed a white precipitate and became homogeneous after approximately 30 min, indicating the formation of the corresponding iminium intermediate. In parallel, 4-hydroxycoumarin (1 equiv.) was dissolved in 5 ml of absolute methanol containing 0.5 ml of 35% hydrochloric acid to promote enolization. This solution was then added dropwise to the iminium intermediate under continuous stirring. The reaction was heated at reflux in an oil bath at 80°C for 15−20 h. Progress was monitored periodically using thin-layer chromatography with chloroform : methanol (2 : 1, v/v) as the eluent. Upon completion, the crude product was recrystallized from methanol and washed with either methanol or ethyl acetate to afford the purified Mannich base.

#### 2.2.2. Mechanism reactions

**Scheme 2.**
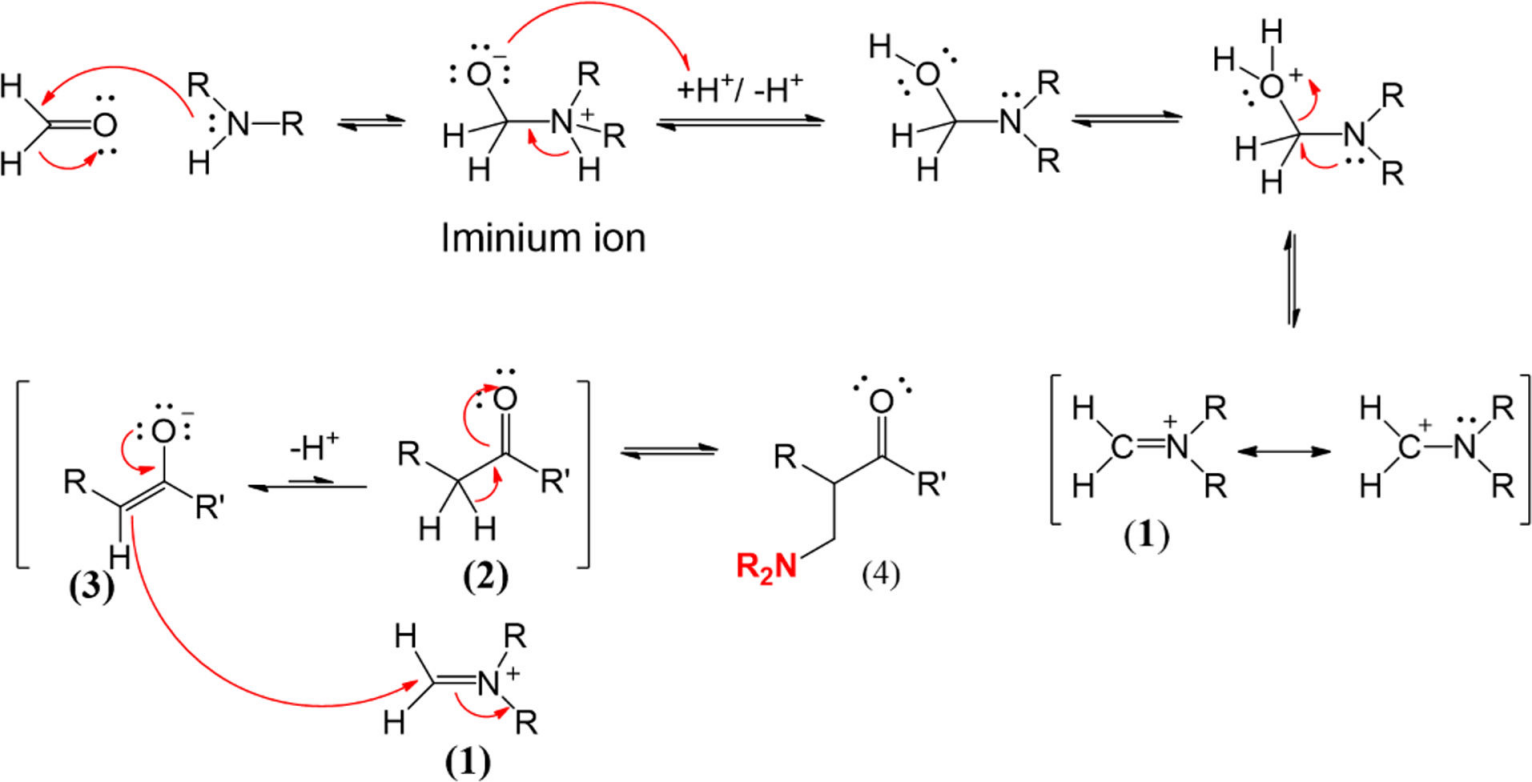
Coumarin derivatives are synthesized via the Mannich reaction mechanism.

The reaction mechanism for synthesizing coumarin derivatives (Scheme 2) proceeds through the following four key stages: (i) nucleophilic attack, (ii) iminium ion formation, (iii) enolate stabilization and cyclization, and (iv) final deprotonation. Initially, the amine attacks the carbonyl carbon, leading to a tetrahedral intermediate that rearranges into a hemiaminal. Carbonyl compounds are widely used in synthetic processes and drug discovery—as intermediates, reagents, pharmacophores, etc. [43–45]. In the second stage, protonation and dehydration convert the hemiaminal into a highly reactive iminium ion, which serves as a crucial intermediate. The third stage involves enolate formation, where resonance stabilization facilitates intramolecular cyclization, driving the formation of the coumarin core. Finally, deprotonation restores aromaticity, yielding the desired coumarin derivative. This well-orchestrated sequence highlights the role of electronic stabilization and proton transfers in constructing biologically relevant coumarin scaffolds [46,47].

#### 2.2.3. Physiochemistry of compounds

**4-Hydroxy-3-[(methylamino)methyl]-2*H*-chromen-2-one (2a):** yellow solid, yield of 52% Fourier transform infrared (FT-IR) (*λ*_max_, cm^–1^, KBr): 3145, 3079 (CH_3_), 2991 (CH_2_), 2865 (C−H), 1649, 1599 (C=O), 1499, 1452 (C=C), 1348, 1109 (C−O, C−N), 773 (an aromatic ring containing a substituent); ^1^H-NMR (500 MHz, CDCl_3_): δ (ppm) 2.46 (s, 3H, CH_3_), 3.63 (s, 2H, CH_2_), 7.33 (d, *J* = 7.9 Hz, 1H, Ar−H), 7.36 (d, *J* = 8.0 Hz, 1H, Ar−H), 7.70 (d, *J* = 8.0 Hz, 1H, Ar−H), 7.88 (d, *J* = 7.9 Hz, 1H, Ar−H); ^13^C-NMR (125 MHz, CDCl_3_): δ (ppm) 169.7 (C-3, C=O, lactone), 165.1 (quaternary carbon, C-4), 153.6 (CH, C-8), 131.4 (CH, C-7), 124.3 (CH, C-6), 123.8 (CH, C-5), 116.8 (quaternary carbon, C-9), 116.6 (quaternary carbon, C-10), 96.7 (quaternary carbon, C-3), 44.4 (CH_2_), 36.0 (CH_3_); HR-MS (ESI-MS): calculation: [M + OH]^+^ = 222.0766; experiment [M + H]^+^ = 222.0592. The protons of N–H and O–H disappeared because the flexible hydrogens of N−H or O−H bonding are exchanged extremely fast with protons with a residual peak of CHCl_3_ in the nuclear magnetic solvent, CDCl_3_, which the ^1^H-NMR has not detected.

**4-Hydroxy-3-[(isopropyl amino)methyl]-2*****H*****-chromen-2-one (2b):** yellow solid, yield of 55%; FT-IR (*λ*_max_, cm^–1^, KBr): 2981 (CH_2_), 2800 (C−H), 1639 (C=O), 1513, 1458 (C=C), 1326, 1197 (C−O, C−N), 755 (an aromatic ring containing a substituent); ^1^H-NMR (500 MHz, CDCl_3_): δ (ppm) 1.16 (d, *J* = 6.7 Hz, 6H, CH_3_), 2.04 (s, 1H, N−H), 2.84 (sextet, 1H, C**H**, isopropyl group), 3.68 (s, 2H, CH_2_), 7.31−7.37 (m, 2H, Ar−H), 7.70 (t, *J* = 7.5 Hz, 1H, Ar−H), 7.88 (d, *J* = 10.0 Hz, 1H, Ar−H); ^13^C-NMR (125 MHz, CDCl_3_): δ (ppm) 163.7 (C=O, lactone), 162.5 (quaternary carbon, C-4), 152.5 (CH, C-8), 126.6 (CH, C-7), 125.0 (CH, C-6), 124.2 (CH, C-5), 16.9 (quaternary carbon, C-9), 116.6 (quaternary carbon, C-10), 103.0 (quaternary carbon, C-3), 49.4 (CH_2_), 24.5 (CH(**C**H_3_)_2_); HR-MS (ESI-MS): calculation: [M + H]^+^ = 234.1085, experiment: [M + H]^+^ = 234.1122. The proton of O–H disappeared because the flexible hydrogen of O–H bonding has been exchanged very fast with a proton with a residual peak of CHCl_3_ in the nuclear magnetic solvent, CDCl_3_, which the ^1^H-NMR has nodetected.

**4-{[(4-hydroxy-2-oxo-2*****H*****-chromen-3-yl)methyl]amino}benzoic acid (2c):** white solid, yield of 60%, FT-IR (*λ*_max_, cm^–1^, KBr): 3315 (N−H), 2989 (CH_2_), 2727 (C−H), 1624, 1597 (C=O), 1522 (C=C), 1302, 1274 (C−O, C−N), 907, 665 (an aromatic ring containing a substituent); ^1^H-NMR (500 MHz, CDCl_3_): δ (ppm): 8.01−7.97 (m, 3H, Ar−H), 7.63−7.60 (m, 1H, Ar−H), 7.38−7.31 (m, 2H, Ar−H), 6.79−6.75 (m, 2H, Ar−H), 4.01 (t, *J* = 6.8 Hz, 1H, N−H), 3.73 (d, *J* = 6.8 Hz, 2H, CH_2_); ^13^C-NMR (125 MHz, CDCl_3_): δ (ppm) 169.7(C=O, COOH), 168.2 (C=O, lactone), 165.1 (quaternary carbon, C-4), 153.6 (CH, C-8), 148.6, 131.4, 130.3, 124.3 (CH, C-5), 123.8 (CH, C-7), 123.5 (C-6), 116.8 (quaternary carbon, C-9), 116.5 (quaternary carbon, C-10), 112.0, 96.7 (quaternary carbon, C-3), 44.4 (CH_2_); HR-MS (ESI-MS): calculation: [M + H]^+^ = 312.1574, experiment: [M + H]^+^ = 312.2487. The protons of carboxylic acid and O−H disappeared because the flexible hydrogens of COOH or O−H bonding are exchanged very fast with protons with a residual peak of CHCl_3_ in the nuclear magnetic solvent, CDCl_3_, which the ^1^H-NMR has not detected.

**4-{[(4-Hydroxy-2-oxo-2*****H*****−chromen-3-yl)methyl]amino}benzonitrile (2d):** white solid; yield: 57%; FT-IR (*λ*_max_, cm^–1^, KBr): 3315 (N−H), 2989 (CH_2_), 2727 (C−H), 1624, 1597 (C=O), 1522 (C=C), 1274, 1242 (C−O, C−N), 907, 665 (an aromatic ring containing a substituent); ^1^H-NMR (500 MHz, CDCl_3_): δ (ppm) 11.3 (s, 1H, N−H), 8.01−7.26 (m, 8H, Ar−H), 3.84 (s, 2H, CH_2_); ^13^C-NMR (125 MHz, CDCl_3_): δ (ppm): 168.9 (C=O, lactone group), 164.6 (quaternary carbon, C-4), 152.5 (CH, C-8), 132.7 (CH, C-7), 124.9 (CH, C-6), 124.2 (CH, C-5), 116.9 (quaternary carbon, C-9), 116.6 (quaternary carbon, C-10), 103.1 (quaternary carbon, C-3), 20.1 (CH_2_); HR-MS (ESI-MS): calculation: [M + H]^+^ = 293.0926, experiment [M + H]^+^ = 293.0859. The proton of O−H disappeared because the flexible hydrogen of O−H bonding has been exchanged extremely fast with a proton with a residual peak of CHCl_3_ in the nuclear magnetic solvent, CDCl_3_, which the ^1^H-NMR has not detected.

**3-[(1*****H*****-imidazol-1-yl)methyl]-4-hydroxy-2*****H*****-chromen-2-one (2e):** white solid; yield: 62%; FT-IR (*λ*_max_, cm^–1^, KBr): 3064 (N−H), 2730 (C−H), 1649, 1599 (C=O), 1347, 1308 (C−O, C−N), 749, 670 (an aromatic ring containing a substituent); ^1^H-NMR (500 MHz, CDCl_3_): δ (ppm) 4.4 (s, 2H, CH_2_), 8.0 (dd, *J* = 8.6, 1.8 Hz, 1H, Ar−H), 7.76 (t, *J* = 1.7 Hz, 1H, Ar−H), 7.59−7.63 (m, 1H, Ar−H), 7.31−7.38 (m, 2H, Ar−H), 7.13 (dd, *J* = 7.1, 1.0 Hz, 1H, imidazole-H), 6.9 (dd, *J* = 4.4, 1.7 Hz, 1H, imidazole-H); ^13^C-NMR (125 MHz, CDCl_3_): δ (ppm) 163.4 (C=O, lactone), 157.8 (quaternary carbon, C-4), 153.4 (CH, C-8), 118.0 (quaternary carbon, C-9), 124.9 (CH, C-6), 123.1 (CH, C-5), 101.5 (CH, C-3) (137.1 (CH, C-7), 115.9 (quaternary carbon, C-10), 41.7 (CH_2_). HR-MS (ESI-MS): calculation: [M + 2H + 2Na]^+^ = 290.0643, experiment [M + 2H + 2Na]^+^ = 290.0603.

**3-[(Cyclopentylamino)methyl]-4-hydroxy-2*****H*****-chromen-2-one (2f):** white solid, yield of 68%; FT-IR (*λ*_max_, cm^–1^, KBr): 3050 (N−H), 2959 (CH_2_), 2875(C−H), 1600 (C=O), 1538, 1465 (C=C), 1363, 1339 (C−O, C−N), 757 (an aromatic ring containing a substituent) ; ^1^H-NMR (500 MHz, CDCl_3_): δ (ppm) 1.47−1.53 (m, 3H, cyclopentylamine-H), 1.61−1.74 (m, 4H, cyclopentylamine-H), 1.85−1.90 (m, 1H, cyclopentylamine-H), 1.95−1.99 (m, 1H, cyclopentylamine-H), 3.89 (s, 2H, CH_2_), 7.13−7.15 (m, 1H, Ar−H), 7.16−7.22 (m, 1H, Ar−H), 7.41−7.47 (m, 1H, Ar−H), 7.82−7.84 (m, 1H, Ar−H); ^13^C-NMR (125 MHz, CDCl_3_): δ (ppm) 168.9 (C=O, lactone), 164.5 (quaternary, C-4), 152.5 (CH, C-8), 132.7 (CH, C-7), 126.6 (CH, C-6), 125.0 (CH, C-5), 124.2, 121.5, 116.9 (quaternary carbon, C-9), 116.6 (quaternary carbon, C-10), 103.0 (quaternary carbon, C-3), 36.0 (CH_2_), 20.1; HR-MS (ESI-MS): calculation: [M + H]^+^ = 260.1286, experiment [M + H]^+^ = 260.1270.

**4-Hydroxy-3-(morpholinomethyl)-2*****H*****-chromen-2-one (2g)**: white solid, yield of 51%; FT-IR (*λ*_max_, cm^–1^, KBr): 3045, 3079 (CH_3_), 2991 (CH_2_), 2753 (C−H), 1624 (C=O), 1452 (C=C), 1348, 1109 (C−O, C−N), 773 (an aromatic ring containing a substituent); ^1^H-NMR (500 MHz, CDCl_3_): δ (ppm) 11.31 (s, 1H, HO), 8.01 (dd, *J* = 8.0, 1.0 Hz, 1H, Ar−H), 7.56 (t, *J* = 7.5 Hz, 1H, Ar−H), 7.57 (t, 1H, Ar−H), 7.35−7.39 (m, 3H, Ar−H), 4.10 (d, *J* = 11.5 Hz, 4H, morpholinomethyl-H), 3.84 (s, 2H, CH_2_), 2.41 (t, *J* = 6.0 Hz, 4H, morpholinomethyl-H); ^13^C-NMR (125 MHz, CDCl_3_): δ (ppm) 163.8 (C = O, lactone), 161.1 (quaternary carbon, C-4), 152.5 (CH, C-8), 106.3 (quaternary carbon, C-3), 133.3 (CH, C-7), 124.9 (CH, C-6), 122.3 (CH, C-5) 116.9 (quaternary carbon, C-9), 116.7 (quaternary carbon, C-10), 66.6 (CH_2_, morpholyl), 52.29 (CH_2_, morpholyl), 51.5 (CH_2_); HR-MS (ESI): calculation: [M + H]^+^ = 262.1079, experiment [M + H]^+^ = 262.1139.

**4-Hydroxy-3-[(propylamino)methyl]-2*****H*****-chromen-2-one (2h)**: white solid, yield of 59%; FT-IR (*λ*_max_, cm^–1^, KBr): 3232 (N−H), 2966 (CH_2_), 2794, 2638 (C−H), 1648, 1601 (C=O), 1525, 1426 (C=C), 1326, 1200 (C−O, C−N), 752 (an aromatic ring containing a substituent); ^1^H-NMR (500 MHz, CDCl_3_): δ (ppm) 0.91 (t, 3H, *J* = 7.5 Hz, CH_3_), 1.54 (q, 2H, *J* = 7.5 Hz, 2H, CH_2_ propylamine), 2.54 (t, 2H, *J* = 5.7 Hz, CH_2_ propylamine), 3.61 (s, 2H, proton, CH_2_), 7.28−7.39 (m, 4H, Ar−H); ^13^C-NMR (125 MHz, CDCl_3_): δ (ppm) 169.7 (C=O, lactone), 165.1 (quaternary carbon, C-4), 153.6 (CH, C-8), 131.3 (CH, C-7), 124.3 (CH, C-6), 123.7 (CH, C-5), 116.8 (quaternary carbon, C-9), 116.6 (quaternary carbon, C-9), 96.7 (quaternary carbon, C-3), 51.9 (CH_2_ of n-propyl group), 44.4 (CH_2_), 23.4 (CH_2_ of n-propyl group), 11.5 (CH_3_, n-propyl group); HR-MS (ESI-MS): calculation: [M + H]^+^ = 234.1085, experiment value [M + H]^+^ = 234.1022. The protons of N−H and O−H disappeared because the flexible hydrogens of N−H or O−H bonding are exchanged extremely fast with protons with a residual peak of CHCl_3_ in the nuclear magnetic solvent, CDCl_3_, which the ^1^H−NMR has not detected.

**4-{[(4-Hydroxy-2-oxo−2*****H*****-chromen-3-yl)methyl]amino}benzenesulfonic acid (2i)**: white solid, yield of 67%; FT-IR (*λ*_max_, cm^–1^, KBr): 3116 (N−H), 2975 (CH_2_), 1655, 1605 (C = O), 1564 (C=C), 1289 (C−O, C−N), 906 (an aromatic ring containing a substituent); ^1^H-NMR (500 MHz, CDCl_3_): δ (ppm) 7.98 (dd, *J* = 8.2, 1.7 Hz, 1H, Ar−H), 7.61 (dd, *J* = 8.2, 7.5 Hz, 1H, Ar−H), 7.39−7.32 (m, 1H, Ar−H), 7.36−7.29 (m, 3H, Ar−H), 6.91−6.85 (m, 2H, Ar−H), 3.95 (t, *J* = 6.8 Hz, 1H, N−H), 3.73 (d, *J* = 6.8 Hz, 2H, CH_2_); ^13^C-NMR (125 MHz, CDCl_3_): δ (ppm) 163.7 (C=O, lactone), 162.6 (quaternary carbon, C-4), 151.9 (CH, C-8), 147.3 (C-4′, Ar′), 132.9 (CH, C-7), 131.1 (CH, C-1′), 127.1 (CH, C-6), 123.4 (CH, C-5), 121.8 (CH, C-3′), 121.6 (CH, C-2′), 116.9 (quaternary carbon, C-9), 116.4 (quaternary carbon, C-10), 102.4 (quaternary carbon, C-3), 40.0 (CH_2_); HR-MS (ESI-MS): calculation: [M + H]^+^ = 348.0542, experiment [M + H]^+^ = 348.0547. The protons of O−H and SO_3_H group disappeared because the flexible hydrogens of O−H or SO_3_H bonding have been exchanged very fast with a proton with a residual peak of CHCl_3_ in the nuclear magnetic solvent, CDCl_3_, which the ^1^H-NMR has not detected.

**4-Hydroxy-3-[(phenylamino)methyl]-2*****H*****-chromen-2-one (2j)**: white solid, yield of 59%; FT-IR (*λ*_max_, cm^–1^, KBr): 3108 (N−H), 2974 (CH_2_), 1654 (C=O), 1564, 1531 (C=C), 1348 (C−O, C−N), 907 (an aromatic ring containing a substituent); ^1^H-NMR (500 MHz, CDCl_3_): δ (ppm) 7.98 (dd, *J* = 8.2, 1.8 Hz, 1H, Ar−H), 7.61 (dd, *J* = 8.2, 7.5 Hz, 1H, Ar−H), 7.44−7.23 (m, 2H, Ar−H), 7.16−6.95 (m, 2H, Ar−H), 6.81−6.53 (m, 3H, Ar−H), 3.99 (t, *J* = 6.8 Hz, 1H, N−H), 3.73 (d, *J* = 6.8 Hz, 2H, CH_2_); ^13^C-NMR (125 MHz, CDCl_3_): δ (ppm) 164.5 (C=O, lactone), 160.7 (quaternary carbon), 153.4 (CH, C-8), 145.9 (quaternary carbon, C-1′, Ar′), 133.3 (CH, C-7), 129.5 (CH, C-6), 124.9 (CH, C-5), 122.3 (CH, C-3′, Ar′), 119.6 (quaternary carbon, C-9), 118.1 (quaternary carbon, C-10), 115.7 (CH, C-4′, Ar′), 113.5 (CH, C-2′, Ar′), 101.6 (quaternary carbon, C-3), 37.03 (CH_2_); HR-MS (ESI): theory calculation: [M + H]^+^ = 268.0973, experiment value [M + H]^+^ = 268.2843. The proton of O−H disappeared because the flexible hydrogen of O−H bonding has been exchanged extremely fast with a proton with a residual peak of CHCl_3_ in the nuclear magnetic solvent, CDCl_3_, which the ^1^H-NMR has not detected.

All the physico-chemical analysis data of IR, ^1^H, ^13^C-NMR and HR-MS spectra of (**2a**–**2j**) compounds showed that the indicated signals were in the appropriate and correct regions and values. The FT-IR spectra of compounds have shown peaks such as the strong vibration stretching of N−H bonding from 3050 to 3315, except for (**2a**), (**2b**) and (**2g**). The methyl groups are from 3045 to 3079 cm^−1^ in (**2a**) and (**2g**), and the methylene group changed from 2865 to 2991 cm^−1^, except for (**2e**). The stretching vibrations of C–H bonding of phenyl groups appeared from 2638 to 2875 cm^−1^, except for (**2a**) and (**2j**). The stretching vibrations of C=O bonding are in the range of 1597–1655 cm^−1^. The stretching vibrations of C=C bonds varied from 1426 to 1564, except for (**2e**). The stretching vibrations of C−N or C−O single bond are in the range of 1109–1363 cm^−1^, and aromatic bearing substitutions are 665–907 cm^−1^. For ^1^H-NMR spectra of (**2a**–**2j**), the resonance signals of N−H protons have varied from 2.04 to 11.3 ppm, except for (**2a**) and (**2e**−**2h**). The methylene protons are maintained from 3.61 to 4.44 ppm for all compounds that proved successful synthesis. The aromatic protons have appeared from 6.53 to 8.01 ppm. For ^13^C-NMR, the resonance signals of carbons of the methylene group have been observed in the range of 36.0–51.5 ppm, except for (**2d**), and the signal of the methylene group appeared at 20.1 ppm. The chemical shifts of lactone carbons resonated in the range of 163.4–169.7 ppm. The signals of quaternary carbons such as C-3, C-4, C-9 and C-10 have resonated in the range of 96.7–106.3, 157.8–165.4, 116.8–119.6 and 115.9–118.1 ppm, respectively. The signals of CH carbons of C-5–C-8 have indicated that the chemical shifts are 122.3–125.0, 123.4–129.5, 123.8–137.1 and 151.9–153.6, respectively.

#### 2.2.4. Antimicrobial activity

All coumarin derivatives were evaluated for *in vitro* antibacterial and antifungal activities, and the minimum inhibitory concentration (MIC) was ascertained in µM using the broth microdilution method. The test organisms were cultivated and cultured for 48 h at 37°C. The 2-day-old bacterial cultures were emulsified in a small volume of Muller Hinton broth (MHB) and cultured at 37°C overnight to reach the logarithmic growth phase. The turbidity of each inoculum was subsequently corrected to McFarland standard no. 0.5 by the additional incorporation of MHB, resulting in an inoculum concentration of 1.5 × 10^8^ CFU ml^−1^. One hundred microlitres of MHB were dispensed into all wells of the 96-well microtiter plates, excluding the first column A. Subsequently, 200 µl of the working solution for each examined chemical was allocated to column A of each plate. A serial twofold dilution was performed beginning with column A, spanning concentrations of 3.91−250 µg.ml^−1^, and the final 100 µl of the working solution was dispensed from the last well. Subsequently, each well was inoculated with 100 µl of the respective bacterial inoculum. Each plate was sealed with parafilm and incubated at 37°C for 24 h. Fifty microliters of 3-(4,5-dimethyl-2-thiazolyl)-2,5-diphenyl-2*H*-tetrazolium bromide (MTT) (0.2 mg ml^−1^ in distilled water) were introduced into each well, followed by incubation of the plates for 30 min at 37°C. Penicillin served as a positive control, while dimethyl sulfoxide functioned as a negative control. Each chemical and medication underwent testing in triplicate on two occasions. The colour transition of MTT from yellow to purple after 30 min signified the existence of viable bacterial cells. The MIC was determined as the minimal concentration of substances that inhibited the colour change [48].

#### 2.2.5. 1,1-Diphenyl−2-picrylhydrazyl assay

The 1,1-diphenyl−2-picrylhydrazyl (DPPH) assay is a technique employed to assess the antioxidant potential of a chemical by measuring its efficacy in scavenging free radicals. DPPH produces stable free radicals in methanol [49]. Upon exposure to an antioxidant, DPPH experiences a colour transformation from purple to yellow, which is quantified using an ELISA reader at a wavelength of 517 nm. The experiment is performed in a 96-well plate, where a 150 µM DPPH solution in 80% methanol is combined with test samples at different concentrations. The DPPH radical scavenging activity percentage is computed using the formula SC% = 1 − [ODt/ODc] × 100 (%), where SC_50_ (radical scavenging concentration of 50%) is the sample concentration necessary to eliminate 50% of DPPH radicals, as ascertained from the standard calibration curve. Trolox serves as a positive control in the experiment.

#### 2.2.6. Sulforhodamine B (SRB) assay

The experiment was conducted as previously outlined, with certain variations [50]. Cells were seeded at a density of 10 000 cells per well (MCF-7, Hep G2, HeLa) or 7500 cells per well (NCI-H460) in 96-well plates, grown for 24 h, and subsequently treated with compounds at varying doses for 48 h. Treated cells were fixed with cold 50% (w/v) trichloroacetic acid (Merck) solution for 1−3 h, subsequently washed and stained with 0.2% (w/v) SRB (Sigma) for 20 min. Following five washes with 1% acetic acid (Merck), the protein-bound dye was solubilized in a 10 mM Tris base solution (Promega). Optical density values were measured using a 96-well microtiter plate reader (Synergy HT, Biotek Instruments) at wavelengths of 492 and 620 nm. The growth inhibition percentage (Inh %) was determined using the formula: Inh % = (1 − [ODt/ODc]) × 100, where ODt and ODc represent the optical density values of the test and control samples, respectively. Camptothecin served as the positive control.

#### 2.2.7. *In silico* molecular docking, molecular dynamic simulation and absorption, distribution, metabolism, excretion and toxicity

*In silico* molecular docking has been performed based on electronic supplementary material, Scheme S1 for docking one ligand to one enzyme by using the AutoDock tools software. To explain the high antibacterial activity of the compound, docking was carried out using the 2VF5 enzyme [51]. The grid parameters include a spacing of 0.400, a grid size of 50 × 50 × 50 along the X, Y, and Z axes, and the coordinates of the active centre (26.577, 22.697, 8.113), which were set up in the grid.gpf file [52]. The docking parameters are 1000 models. Docking was performed using the 1T8I enzyme to account for the compound’s anti-cancer activity observed *in vitro* [51]. The grid parameters are determined by spacing, the number of elements on X, Y and Z axes and the active centre 0.500, (50, 50, 50) and (22.669, 1.499, 30.147), respectively. For studying the *in silico* antioxidant activity, the 1HD2 enzyme has been used to calculate docking, with grid parameters being spacing, the number of elements on X, Y, Z and the active centre being 0.5, 50, 50, 50 and 7.937, 42.687, 20.122. The simulation of the best docking pose 232, the best conformation among 1000 conformation dockings and the crystal structure of 1T8I enzyme had been performed following electronic supplementary material, Scheme S2. The pharmacokinetic or ADMET had been conducted via a web platform by ADMETlab 2.0 and had been calculated for the compound **2d**, which is the best candidate among compounds (**2a**–**2j**) against the human breast cancer cell line MCF-7, an excellent *in vitro* anti-cancer compound.

## 3. Results and discussions

### 3.1. Anti-cancer activity

**Figure 1.**
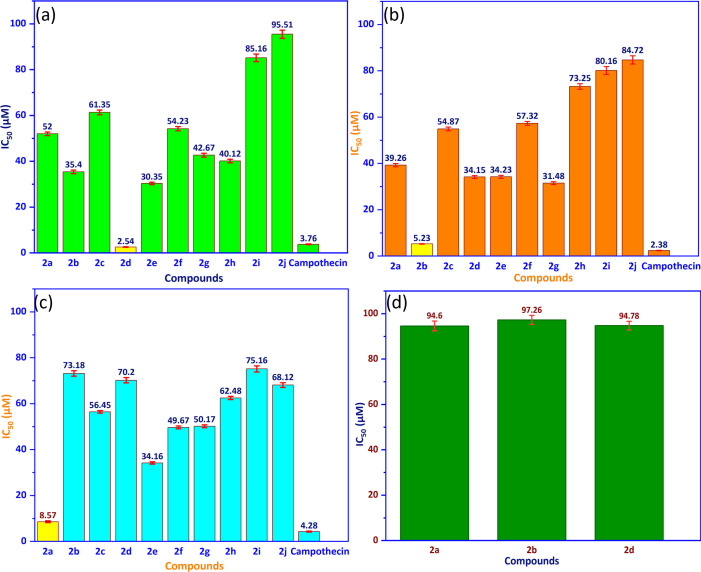
The IC_50_ values (µM) for coumarin derivatives (**2a**–**2j**) were assessed on cell lines: (a) MCF−7 cell line; (b) HeLa cell line; (c) Hep G2 cell line; (d) normal cell (LLCPK1).

**Figure 2.**
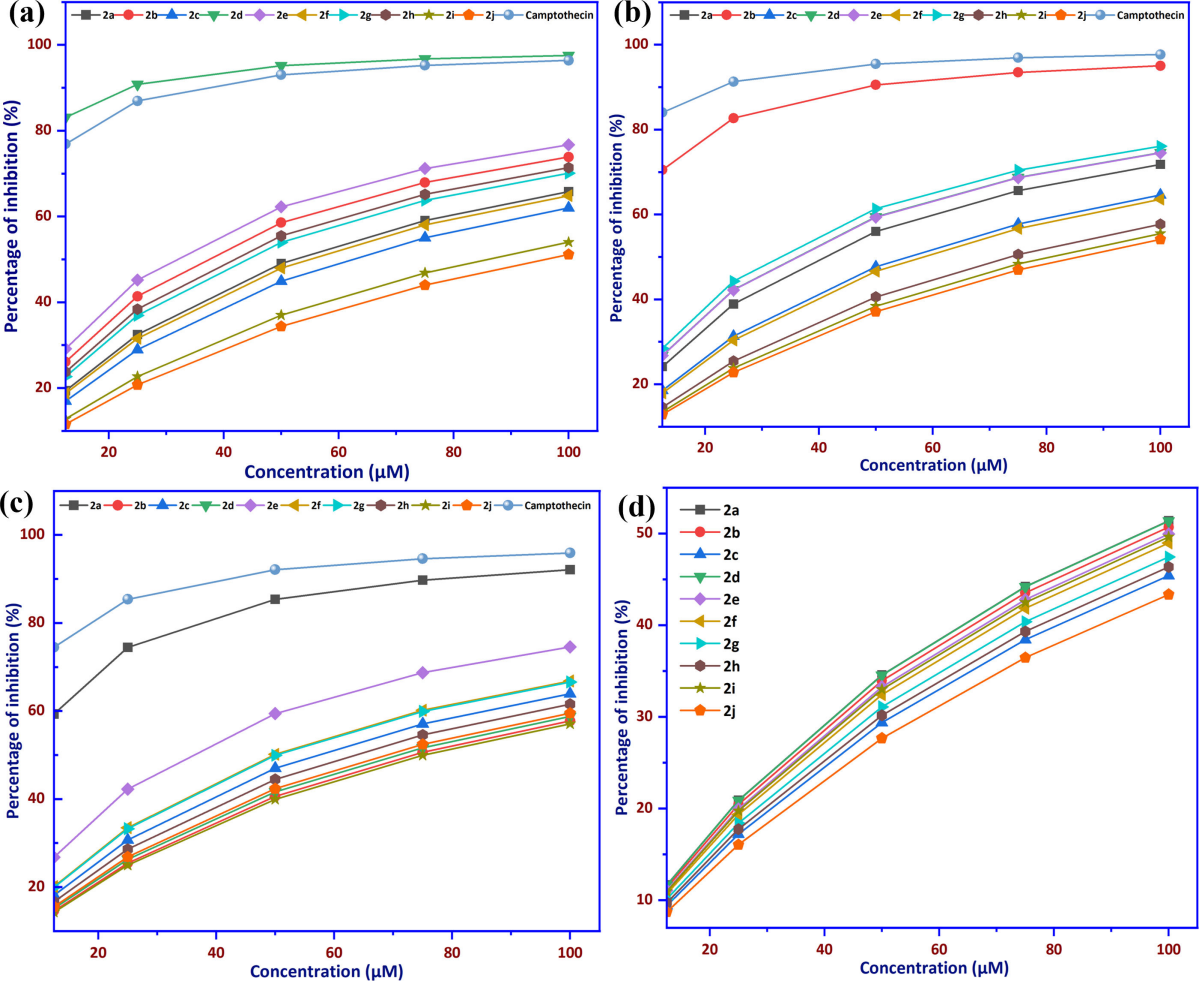
The percentage of inhibition of coumarin derivatives (**2a**−**2j**) against cancer cell lines, respectively, (a) MCF−7, (b) HeLa, (c) HepG2 and (d) normal cells (LLCPK1) *in vitro* in the concentration range of 12.5−100 µM.

The target compounds (**2i**−**2j**) were tested for cytotoxicity by SRB assay, and the inhibition ability of entries (**2i**−**2j**) and drug against cell lines, such as human breast cancer cell line (MCF-7), human cervical cancer cell line (HeLa), human liver cancer cell line (HepG2) and normal kidney epithelial cell line (LLCPK1), is shown in figure 1. The compound **2d** showed pronounced anti-cancer activity against the MCF-7 human breast cancer cell line, with an IC₅₀ value of 2.54 ± 0.12 µM. The cytotoxicity of compound **2d** has been shown to be stronger than that of the drug, with the IC_50_ value of 3.76 ± 0.15 µM. Upon testing the compounds against the human cervical cancer cell line (HeLa), the compound **2b** showed great activity against the HeLa cells, with an IC_50_ value of 5.23 ± 0.12 µM. Compound **2b** has a higher IC_50_ value than the standard drug, which has an IC_50_ value of 2.38 ± 0.102 µM, indicating lower cytotoxicity. The compound **2a** has shown strong activity against the HepG2 cell line of human liver cancer, with the IC_50_ value of 8.57 ± 0.42 µM, which is higher than that of camptothecin (IC_50_ = 4.28 ± 0.201 µM), suggesting lower inhibition. As shown in figure 1d, the cytotoxic effects of the tested compounds were also evaluated in normal kidney epithelial cells (LLCPK1) to assess selectivity. Since compounds **2d**, **2a** and **2b** exhibited significant anti-cancer activity, their cytotoxicity was further tested on normal cells. The IC_50_ values for LLCPK1 were 94.78 µM for the compound **2d**, 94.6 µM for the compound 2a and 97.26 µM for the compound **2b**, indicating minimal toxicity to normal cells. These values are significantly higher than those observed in cancer cell lines, confirming good selectivity. A higher IC_50_ correlates with lower cytotoxicity, meaning weaker inhibition of normal cells, while a lower IC_50_ suggests stronger anti-cancer potential. Among all tested compounds, the IC_50_ values followed the order **2d** < **2c** < **2f** < **2e** < **2g** < **2h** < **2a** < **2b** < **2i** < **2j** in MCF−7; **2b** < **2g** < **2f** < **2e** < **2d** < **2a** < **2h** < **2i** < **2j** in HeLa and **2a** < **2f** < **2e** < **2g** < **2d** < **2c** < **2h** < **2i** < **2j** in HepG2. These differences in IC_50_ suggest that the compounds’ abilities to kill cancer cells depend on how they are structured and how each cancer cell line works biologically. Overall, compound **2d** had the strongest cytotoxic effect on all the cancer cell lines that were tested, while compound **2j** had the weakest inhibition. This shows that there are big differences in how well compounds work. Several studies have investigated the cytotoxicity of coumarin derivatives against cancer cell lines. Researchers Ragab *et al.* made new coumarin derivatives and found that compound **9d** was the most effective against MCF-7, with an IC_50_ value of 0.017 µM. This was a lot stronger than the compound **2d**, which had an IC_50_ value of 2.54 ± 0.12 µM. However, their synthesis requires a minimum of three stages or a maximum of five, including condensation reactions and extensive purification, making it more complex than the one-step procedure used in this study [53]. Citarella *et al.* found compound 210 to be a BRD4 inhibitor with IC_50_ values of 2.01 µM for MCF-7 and 4.76 µM for HepG2. This suggests that the compound 210 has similar effects as compounds **2d** and **2a** in this study. However, their synthetic route had many steps, such as coupling reactions and functional group modifications that had to be done under strict conditions. This made the approach even simpler in this study [54]. According to Gomaa *et al.*, compounds **4k** and **6c** had IC_50_ values of 4.98 and 5.85 µM for MCF-7 and 9.4 and 33.88 µM for HepG2 [55]. This means that the compound **2d** was more effective against MCF-7, while compound **2a** was the same as compound **4k** in HepG2. Their method has at least three steps that include strategies for protecting and deprotecting molecules, as well as a lot of cleaning up. This study shows that the one-step synthesis is a more efficient and useful way to get coumarin derivatives. These comparisons show that, while some coumarin derivatives were more effective in earlier studies, compounds **2d** and **2b**, in this study, were just as good or better at killing breast and cervical cancer cells. Moreover, their minimal toxicity towards normal cells suggests promising selectivity as anti-cancer agents. The inhibition abilities of all compounds (**2a**–**2j**) at concentrations of 100, 50, 25, 12 and 5 µM against cancer cell lines (MCF-7, HeLa, HepG2) and a normal cell line (LLCPK1), as determined by the SRB assay, are presented in figure 2. As shown in figure 2, all test compounds (**2a**−**2j**) at concentrations ranging from 12.5100 µM have proved their inhibition abilities against three cancer cell lines and a normal kidney epithelial cell line (LLCPK1), generally evaluated in order of MCF-7 > HeLa > HepG2 > LLCPK1 *in vitro*.

### 3.2. Antimicrobial activity

**Figure 3.**
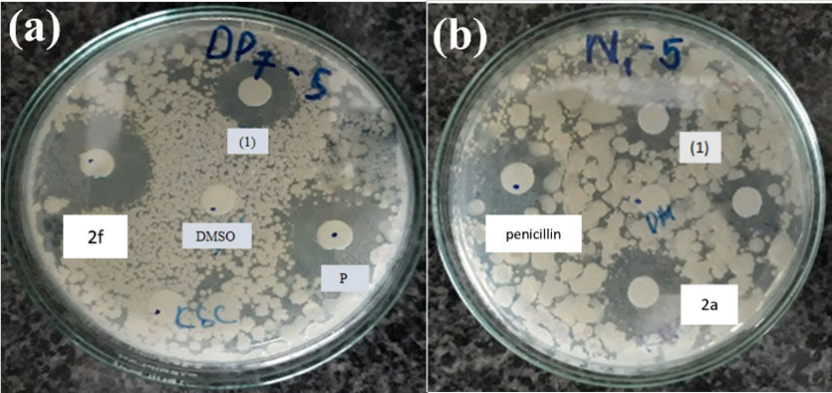
The antibacterial efficacy of compounds (**2a**) and (**2f**) against *B. subtilis.*

The test compounds (**2a**–**2j**) were screened against three bacteria, *E. coli, MRSA* and *B. subtilis*, as well as two fungi, *C. albicans* and *T. mentagrophytes*. Among the test compounds (**2a**–**2j**), only two compounds exhibited antibacterial activity against *B. subtilis* bacteria. The results demonstrated that only compounds **2**a and **2f** exhibited significant inhibitory activity against *B. subtilis*, with MIC values of 25 and 75 µM, respectively, as shown in figure 3. Other compounds did not show any *in vitro* antimicrobial activity against fungi and bacteria.

### 3.3. Antioxidant activity

**Figure 4.**
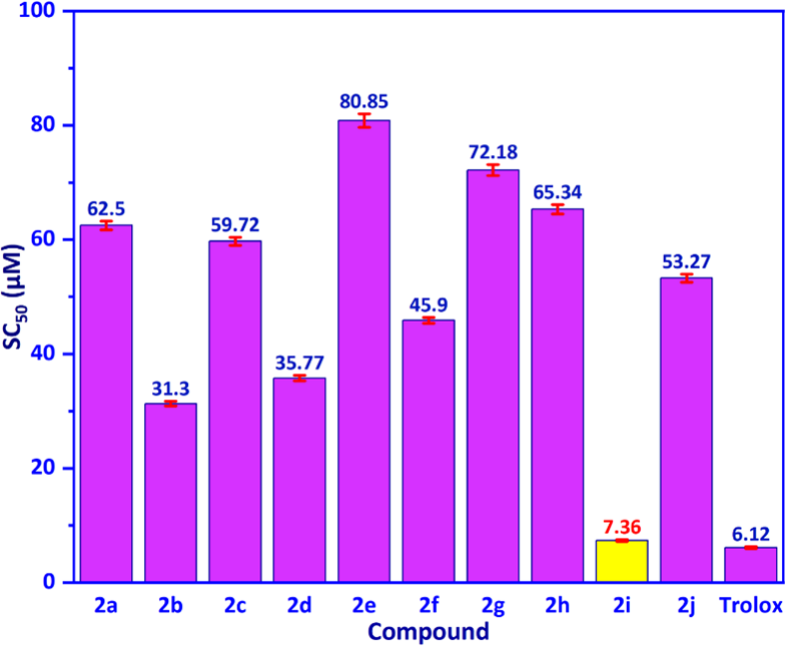
The SC_50_ values (µM) for coumarin derivatives.

The test compounds (**2a**–**2j**) were evaluated for antioxidant activity via DPPH assay, with results presented in figure 4. The SC_50_ values are shown on the *Y*-axis in units of µM against test compounds (**2a**–**2j**) on the X-axis. The compound **2i** demonstrated the highest antioxidant activity among the test compounds, with the SC_50_ value of 7.36 ± 0.20 µM, which is lower than that of the standard drug Trolox, with the SC_50_ value of 6.12 ± 0.15 µM.

### 3.4. *In silico* docking and molecular dynamic simulations

#### 3.4.1. *In silico* docking

##### 3.4.1.1. Anti-cancer *in silico* docking model

**Figure 5.**
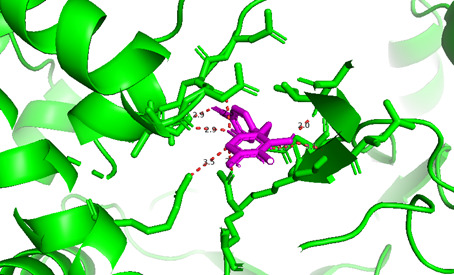
Hydrogen bonding from the active site on pose 232, the best conformation of compound **2d** among the 1000 conformations of the residual amino acids on the 1T8I enzyme by PyMOL software.

**Figure 6.**
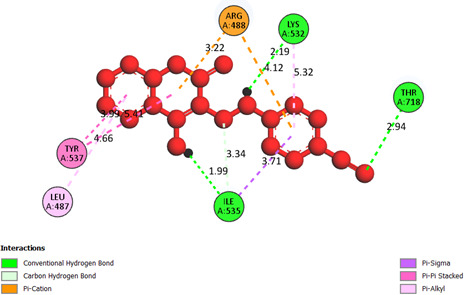
The significant docking results between pose 232 or compound **2d** and the crystal structure of 1T8I: PDB have been presented on a two-dimensional diagram by DSC software.

**Figure 7.**
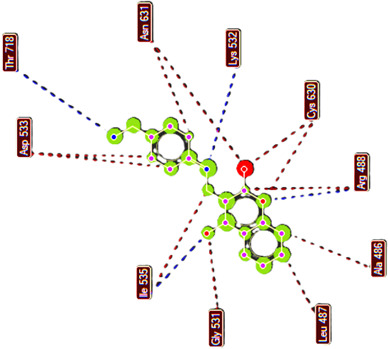
The ligand map presented the secondary interactions between pose 232 or compound **2d** and 1T8I on a two-dimensional diagram.

**Compound 2d, or pose 232**, one of the best docking poses among 1000 docking conformations, has been docked on the active centre on the 1T8I: PDB enzyme, with the values of the free Gibbs energy and inhibition constant, Ki, of −9.62 kcal mol^−1^ and 0.09 µM, respectively, as shown in the electronic supplementary material, table S1. The compound formed six hydrogen bonds with the 1T8I enzyme that is relative to residual amino acids on 1T8I, such as Arg 488, Gly 531, Thr 718, Ile 534 and Lys 532, as shown in the electronic supplementary material, table S1. The high number of hydrogens indicates that pose 232 exhibits greater polarity. PyMOL analysis of the number of hydrogen bonds further confirmed that pose 232 exhibits greater polarity. PyMOL software demonstrated the number of hydrogen bonds within a 5 Å range around pose 232 (violet), as shown in figure 5. As shown in figure 6, pose 232 interacted well with the 1T8I enzyme because three parts of this pose have shown full interactions with the 1T8I enzyme, such as capping group (protein identification part), linker part and functional group [56]. In pose 232, hydrogen bonds were observed between Ile535 and the hydroxyl hydrogen, Thr718 and the nitrile nitrogen, and Lys 532 and the N–H hydrogen. The capping group is pi–pi stacked (pink colours) from Tyr 537 to the pi electron system of the phenyl group and the conjugation pi electrons of the lactone group on pose 232, pi–alkyl interaction from Leu 487 to the pi electron system of the phenyl group, pi–sigma interaction from His 632 to the pi electron system of the phenyl group and pi–cation interaction from Arg 488 to the pi electron system. The linker part or connecting unit has interactions such as pi–cation from Arg 488 to the conjugation pi electrons of the lactone group and van der Waals from Ile 535 to methylene group on pose 232. Pose 232 exhibits both hydrophilic and hydrophobic interactions with the active center of the 1T8I enzyme (chain A). As indicated in figure 7, a two-dimensional interaction presented a ligand map that indicated the secondary interactions, such as hydrogen bonding, steric interactions and overlap interactions, have formed from residual amino acids of the 1T8I enzyme and atoms on pose 232. The interactions proved the processing of pose 232 in the whole docking process. The hydrogen bonding, blue colours, are from Thr 718, Lys 532, Arg 488 and Ile 535 to the active atom on pose 232. The steric interactions, brown colours, are relative to Ile 535, Asp 533, Thr 718, Asn 631, Cys 630, Arg 488, Ala 486, Leu 487, Gly 531 and Ile 535 that are linked to active atoms on pose 232. The overlap interactions are characterized by the diameter of green circles on the atoms of pose 232. The larger diameter of the green circles demonstrated that pose 232 interacted strongly with the 1T8I enzyme throughout the docking process.

**Figure 8.**
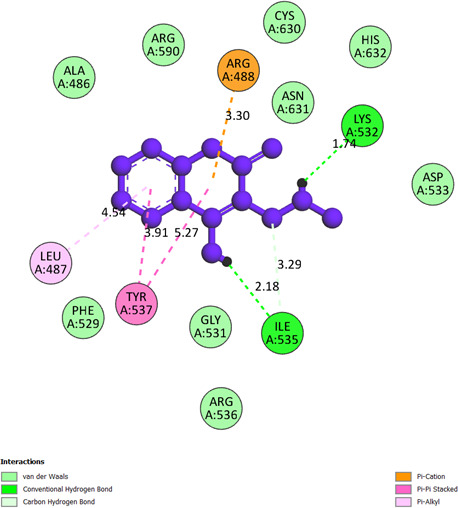
The significant docking results between pose 376 or compound **2a** and the crystal structure of 1T8I: PDB have been presented on a two-dimensional diagram by DSC software.

**Compound 2a or pose 376:** pose 376 has been linked to 1T8I with the values of free Gibbs energy and inhibition constant of −6.2 kcal mol^−1^ and 28.37 µM**,** respectively. It formed five hydrogen bonds with 1T8I via active residual amino acids Arg 488, Gly 531, Ile 535 and Lys 532, as shown in the electronic supplementary material, table S1. It proved that compound **2a** is more hydrophilic. Among the hydrogen bondings, the hydrogen bonding that formed from pose 376 to the enzyme is the strongest because of the shortest bonding length, 1.74 Å. As shown in figure 8, the significant interactions between pose 376 and 1T8I are presented on a two-dimensional diagram. The pose 376 interacted well with 1T8I because the three parts of this pose have shown full ligand interactions. The functional group has formed hydrogen bonding from Lys 532 and Ile 535 to the hydrogen atom of the N−H group and the hydrogen atom of the hydroxyl group, respectively. The capping group of the pose 376 has pi–pi stacked interactions from Tyr 537 to the pi electron system of the phenyl group and the conjugation pi electrons of the lactone group, as well as a pi–cation interaction from Arg 488 to the conjugation pi electrons of the lactone group. The linker part is detected via pi–alkyl interaction from Leu 487 to the pi electron system of the phenyl group and van der Waals from Ile 535 to the methylene group on this pose.

**Figure 9.**
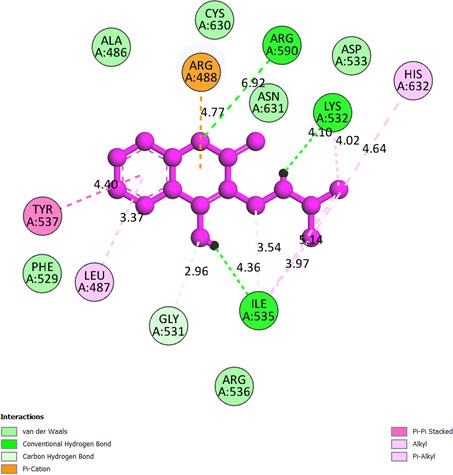
The significant docking results between pose 98 or compound **2b** and the crystal structure of 1T8I: PDB have been presented on a two-dimensional diagram by DSC software.

**Compound 2b or pose 98:** pose 98 anchored well to 1T8I with Gibbs free energy and inhibition constant values of −6.96 kcal mol^−1^ and 7.19 µM, respectively, as indicated in the electronic supplementary material, table S1. It has formed five hydrogen bonds with the active atom in this pose, as shown in the electronic supplementary material, table S1. Among the five hydrogen bondings, the hydrogen bonding from pose 98:H to A:Lys 532:O is the strongest because of the shortest bonding length. The important ligand interactions between pose 98 and residual amino acids on the pose have been demonstrated on a two-dimensional diagram in figure 9. The hydrogen bonding is between Arg 590, Lys 532 and Ile 535 to the oxygen of the lactone group, the hydrogen atom of the N−H group and the hydrogen atom of the OH group, respectively. The protein identification part, or capping group, is pi–pi stacked from Tyr 537 and has one pi–alkyl interaction from Leu 487 to the pi electron system of the phenyl group, and one pi–cation interaction from Arg 488 to the conjugation pi electrons of the lactone group. The linker parts are identified via pi–alkyl interactions from Lys 532, His 632 and Ile 535 to the methyl group, and the carbon–hydrogen interactions from Ile 535 and Gly 531 to the methylene group and the oxygen atom of the hydroxyl group, respectively. Pose 98 interacted well with 1T8I.

**Figure 10.**
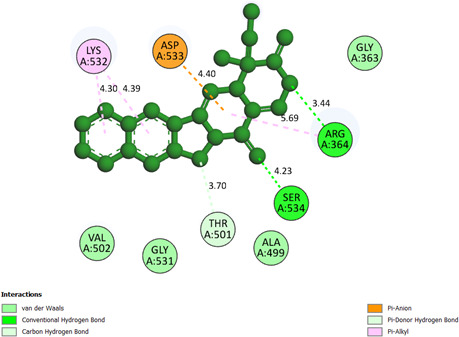
The significant interactions between pose 668 or camptothecin and the 1T8I enzyme on a two-dimensional diagram.

**Camptothecin or pose 668:** pose 668 interacted with the 1T8I enzyme with the values of free Gibbs energy and inhibition constant of −7.72 kcal mol^−1^ and 2.58 µM, respectively. This pose formed two hydrogen bonds from the active atom on the pose to Arg 364 and Ser 534 on the 1T8I enzyme, as shown in the electronic supplementary material, table S1. The significant interactions between residual amino acids on the pose and active atoms on camptothecin are exposed on a two-dimensional diagram, as indicated in figure 10. The pose 668 interacted well with the enzyme because three parts of this pose have shown full ligand interaction. In the pose, Ser534 formed a hydrogen bond with the ketone oxygen, while Arg364 formed a hydrogen bond with the lactone oxygen. The capping group of the pose is discovered by the pi–alkyl interactions from Lys 532 to the conjugation pi electron system of the phenyl group. The linker part is identified via one pi–alkyl interaction from Arg 364, one pi–cation from Asp 533 to the system of conjugation pi electrons of the amide group and one carbon–hydrogen bond interaction from Thr 501 to the carbon atom of the ring.

Pose 232 of compound **2d** has an interaction similar to pose 668 of camptothecin via one pi–alkyl interaction from Lys 532 to the pi electron system of the phenyl group on pose 232 (compound **2d**) or pose 668 (camptothecin).

##### 3.4.1.2. *In silico* antibacterial activity

**Figure 11.**
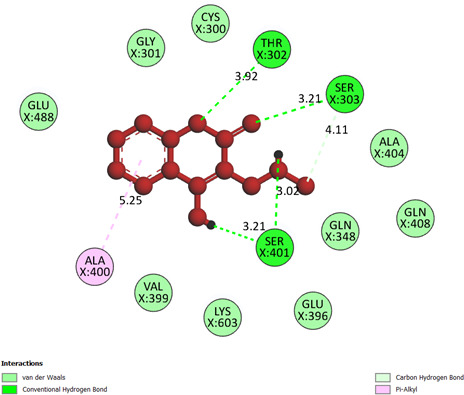
The significant docking results between pose 97 or compound **2a** and the crystal structure of 2VF5: PDB have been presented on a two-dimensional diagram by DSC software.

**Compound 2a or pose 97:** pose 97 interacted with 2VF5 enzyme with the values of free Gibbs energy and inhibition constant of −7.00 and 7.4 µM, respectively. This pose has formed 10 hydrogen bonds with residual amino acids on the 2VF5 enzyme, as shown in the electronic supplementary material, table S1. Among them, the hydrogen bonding that linked from hydrogen on pose 97 to the oxygen atom of Ser 401 is the strongest because of the shortest bonding length, 1.85 Å, as indicated in the electronic supplementary material, table S1. This pose is highly hydrophilic, as it forms a total of 10 hydrogen bonds. The main interactions between pose 97 and 2VF5 are indicated on a two-dimensional diagram, as shown in figure 11. Pose 97 interacted well with 2VF5 because three parts of pose 97 interacted well with 2VF5. In pose 97, Thr302, Ser303, and Ser401 form hydrogen bonds with active atoms of the pose. The capping group of pose 97 is identified via one pi–alkyl interaction from Ala 400 to the system of pi electrons of the phenyl group on the pose. The connecting unit, or linker part, of the pose is observed via one carbon–hydrogen bond from Ser 303 to the methyl group on the pose.

**Figure 12.**
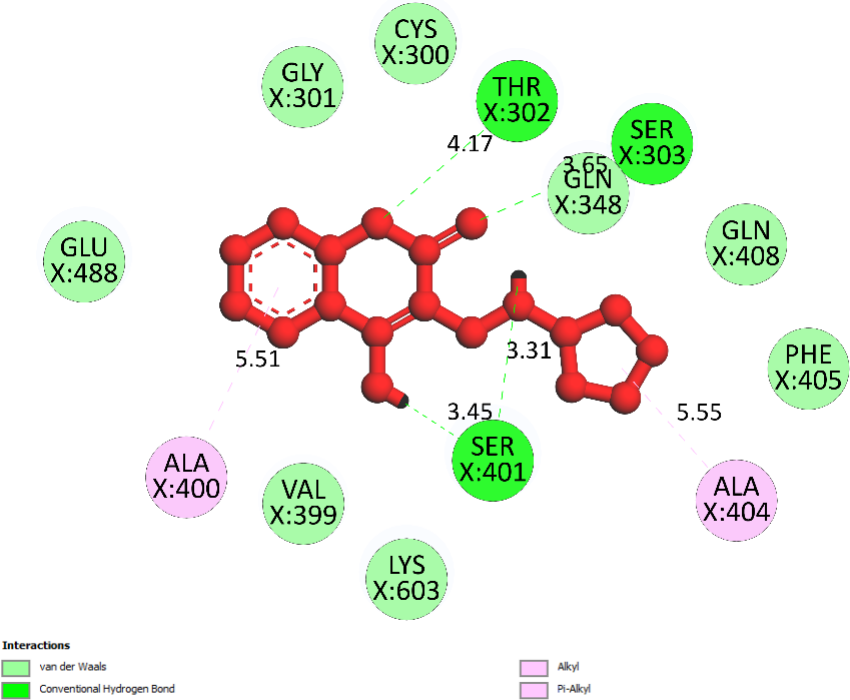
The significant docking results between pose 97 or compound **2f** and the crystal structure of 2VF5: PDB have been presented on a two-dimensional diagram by DSC software.

**Compound 2f or pose 97:** pose 97 interacted with the 2VF5 enzyme with the values of free Gibbs energy and inhibition constant of −7.59 kcal mol^−1^ and 2.74 µM, respectively. It has formed six hydrogen bonds with residual amino acids on 2VF5, such as Thr 302, Ser 303, Ser 401 and Ser 401, as shown in the electronic supplementary material, table S1. Among them, the hydrogen bonding from pose 97 to the oxygen atom of Ser 401 is the strongest because of the shortest bonding length. As shown in figure 12, the significant interactions between pose 97 and 2VF5 are presented on a two-dimensional diagram. Pose 97 docked well to the enzyme because three parts of this pose indicated full interactions. The pose forms four hydrogen bonds: between Ser401 and the hydrogen atom of the OH group, Ser401 and the hydrogen atom of the N–H group, Thr302 and the oxygen atom of the lactone group, and Ser303 and the oxygen atom of the ketone group. The capping group is discovered by one pi–alkyl or alkyl interaction from Ala 400 to the system of pi electrons of the phenyl group. The linker part indicated one pi–alkyl interaction from Ala 404 to the cyclopentyl group of pose 97.

**Figure 13.**
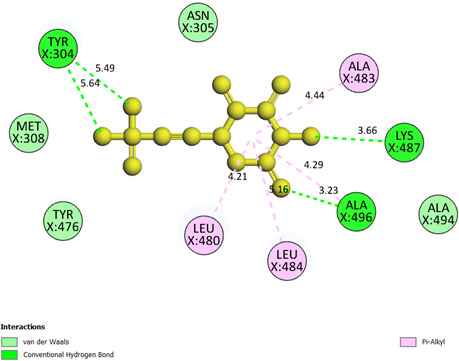
The significant docking results between pose 439 or small ligand in the enzyme and the crystal structure of 2VF5: PDB have been presented on a two-dimensional diagram by DSC software.

**Small ligand in 2VF5 or pose 439:** pose 439 contains a small ligand, which is also present in PDB entry 2VF5. The ligand was redocked into 2VF5 to evaluate the interactions between the pose and the receptor. It anchored to 2VF5 with the values of free Gibbs energy and inhibition constant of −2.77 kcal mol^−1^ and 9.34 mM, respectively. It linked four hydrogen bonds from this pose to 2VF5, as shown in the electronic supplementary material, table S1. The significant docking results between pose 439 or small ligand in the enzyme and the crystal structure of 2VF5: PDB have been presented on a two-dimensional diagram, as shown in figure 13. This pose interacted well with 2VF5 because three parts of this pose have shown full interactions. The functional group of the pose formed four hydrogen bonds from Tyr 304 to two oxygen atoms on the hydroxyl groups of pose 439, Lys 487 to the nitrogen atom of the amino group of pose and Ala 496 to the oxygen atom of the ketone group. The linker and capping group are pi–alkyl interactions that formed from Leu 484, Leu 480, Ala 496 and Ala 483 to the cyclohexyl group on pose 439. Pose 439 is considered a good interaction with enzyme 2VF5.

##### 3.4.1.3. *In silico* antioxidant activity

**Figure 14.**
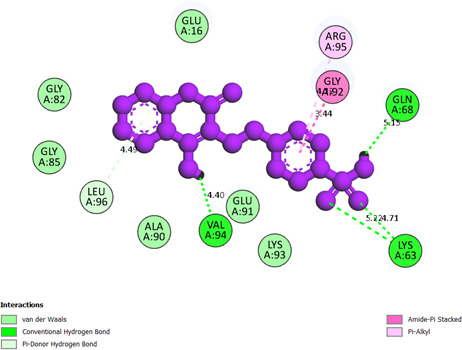
The significant docking results between pose 144 or compound **2i** and the crystal structure of 1HD2: PDB have been presented on a two-dimensional diagram by DSC software.

**Compound 2i, or pose 144**, has anchored to the 1HD2 enzyme with the values of free Gibbs energy and inhibition constant of −9.08 kcal mol^−1^ and 0.22 µM, respectively. This pose formed six hydrogen bonds with 1HD2 that are relative to residual amino acids on 1HD2, such as Lys 63, Glu 91, Val 94 and Gln 68 of the A chain. Among the hydrogen bonding, the hydrogen bonding that formed from Val 94 to pose 144 is the strongest bonding because of the shortest bond length, 1.74 Å, as shown in the electronic supplementary material, table S1. As shown in figure 14, the significant ligand interactions between pose 144 and 1HD2 have been demonstrated on a two-dimensional diagram that indicated pose 144 interacted well with 1HD2. The functional group of pose 144 has four hydrogen bondings from Val 94 to the hydrogen atom of the hydroxyl group, Lys 63 to the oxygen atoms of the S=O bonding of the SO_3_H group and Gln 68 to the hydroxyl atom of the OH group. The linker part has been identified via conventional hydrogen bonding from Leu 96 to the system of conjugation pi electrons of the phenyl group. The capping group, or protein identification part, has pi–alkyl interaction from Arg 95 to the system of conjugation pi electron of the phenyl group and amide pi-stacked from Gly 92 to the system of conjugation pi electrons of the phenyl group. Pose 144 has discovered good interaction with the 1HD2 enzyme.

### 3.4.2. Molecular dynamics simulations

**Figure 15.**
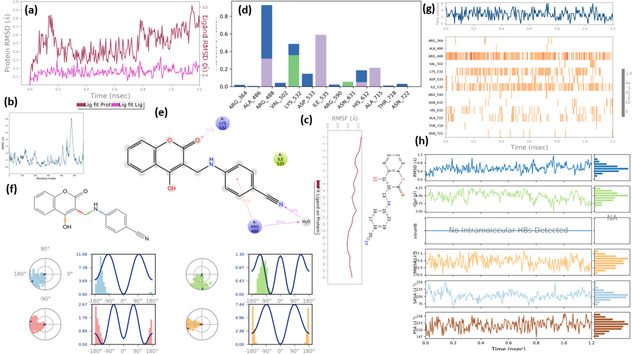
The molecular dynamics (MD) simulation of the complex of pose 232 or compound **2d** in water media via Desmond–Schrodinger software, Linux operating system: (a) plot of RMSD of ligand and 1T8I enzyme, (b) RMSF of 1T8I enzyme, (c) L-RMSF, (d) 1T8I and pose 232 contacts, (e) schematic of detailed pose 232 atom interactions with the amino acid residues on 1T8I, (f) pose 232 torsion, (g) 1T8I-pose 232 contacts via trajectory from 0 to 100 ns and (h) the properties of pose 232.

**Compound 2d, or pose 232,** one of the best docking poses of compound **2d** among 1000 docking conformations, has docked well to the 1T8I enzyme *in vitro* to explain why compound **2d** has shown excellent anti-cancer activity against human breast cancer cell lines, MCF-7 code *in vitro* (**2d***,* IC_50_ of 2.54 µM, MCF-7)*.* This pose has been conducted to make the simulation model by Desmond software in a Linux environment (Linux/Ubuntu operating system), as shown in figure 15a–h. The simulation details have been determined for the 1T8I enzyme, which are constant number of particles, pressure, and temperature (NPT) method, 300K, 1–100 ns, 91 734 atoms, 27 356 water and 0 charge. For pose 232, or ligand **2d**, it has 34 atoms and 22 heavy atoms; the number of rotations bonding is 4. The concentrations of sodium chloride are 66.464 mM of Na^+^ and 50.512 mM of Cl^−^. The numbers of atoms are 76 atoms of Na^+^ and 100 atoms of Cl^−^. As shown in figure 15a, the 1T8I RMSD (left Y-axis) and two-dimensional RMSD (right Y-axis) were plotted over the simulation time (ns, X-axis), with values ranging from 0.1 to 0.8 Å, where the dark red plot indicates minimal conformational changes throughout the simulation. The curve of RMSD of pose 232 has been changed from 0.1 to 0.3 Å, a pink colour plot that has proved the less conformation of these 232 whole simulation times. As shown in figure 15b, the root mean square fluctuation (RMSF) plot of 1T8I was constructed against the residue index, indicating that the amino acid residues varied significantly between indices 450 and 500. The RMSF is useful for characterizing local changes along the protein chain. As shown in figure 15c, pose 232 has been fitted on 1T8I, which is characterized by the values of RMSF of pose 232. The ligand RMSF (L-RMSF) is useful for characterizing changes in the ligand atom, and the C-1 atom has the largest fluctuation with the value of RMSF more than 1.5 Å. As shown in figure 15d, the enzyme interactions with the ligand can be monitored throughout the simulation. These interactions have been categorized into different types and summarized, as shown in the plot above. Enzyme–ligand interactions (or ‘contacts’) are categorized into four types: hydrogen bonds, hydrophobic, ionic and water bridges. Each interaction type contains more specific subtypes, which can be explored through the ‘simulation interactions diagram’ panel. Arg 488 amino acid interacted with pose 232, controlled over 90 ns via water bridges, which is a hydrophobic interaction. The Lys 532 amino acid hydrogen interaction with pose 232 via a water bridge is maintained for 50 ns. Ile 535 is bound to pose 232 via one hydrophobic interaction that controlled 60 ns. Other amino acids are relative to interactions with pose 232 at less time, such as His 632 (30 ns), Ala 715 (25 ns) and Asp 533 (10 ns). As shown in figure 15e, the schematic of pose 232 interactions with the amino acid residues of 1T8I shows that Lys 532 interacted with the oxygen atom of the ketone group for more than 35% of the simulation time, representing a positively charged interaction. As indicated in figure 15f, the torsion profile of pose 232 was determined to include four types of torsional bonds: C–coumarin ring–OH (orange), C–coumarin–CH_₂_ (pink), C–phenyl–NH (blue), and CH_₂_–NH (green). Among the torsional bonds, C–phenyl–NH (blue) exhibited the greatest torsional effect, with torsion energy ranging from 3.69 to 11.08 kcal mol⁻¹, corresponding to maximum torsion angles of −90° and +90°. As indicated in figure 15g, the 1T8I-pose 232 contacts via trajectory from 0 to 100 ns, the residual amino acids on 1T8I that controlled more interaction times with pose 232 are Arg 488, Lys 532 and Ile 535. As indicated in figure 15h, the properties of pose 232 during the entire simulation process showed that its RMSD (in Å) varied from 0.2 to 1 Å, which supports the validation of the docking model of this pose with the 1T8I enzyme The radius of gyration (*r*_Gyr_) is in the range of 4.0–4.25 Å. The molecule surface area is in the range of 27.25–27.50 Å. The solvent accessible surface area is in the range of 100–125 Å^2^. The polar surface area is in the range of 150–156 Å^2^.

#### 3.4.2.1. Pharmacokinetic prediction: absorption, distribution, metabolism, excretion, and toxicity

**Compound 2d or pose 232:** pharmacokinetics of pose 323 has been calculated and demonstrated in the electronic supplementary material, tables S2–S10. As shown in the electronic supplementary material, table S2, the physico-chemical properties of compound **2d** or pose 232 indicate that all the optimal parameters are in the permissible ranges. The value of log S is −3.158, which proves S is small and the aqueous solubility is less. The value of log P is 2.253, which is greater than zero, indicating that the compound is more soluble in the octanol phase than in the aqueous phase. As indicated in the electronic supplementary material, table S3, the medicinal chemistry parameters of the compound have been demonstrated, and it has been shown that most of the values are optimal values; for instance, Lipinski Rule, Pfizer Rule, GSK Rule and Golden Triangle are accepted based on the standards of drug design. The MCE-18 value is 16, and it is lower than 45, which indicates it as a less suitable value. The Fsp³ value is 0.059, which is lower than the standard value of 0.42, indicating that the melting point and solubility are less suitable. The absorption properties of the compound are within the permissible ranges, as shown in the electronic supplementary material, table S4. The value of Caco-2 cell permeability, the human colon adenocarcinoma cell lines (Caco-2), is −4.396 > −5.15, an optimal value, and it has been proven that the compound is an important index for an eligible candidate drug compound. It is explained that before an oral drug reaches the systemic circulation, it must pass through intestinal cell membranes via passive diffusion, carrier-mediated uptake or active transport processes. As an alternative approach to the human intestinal epithelium, it has been commonly used to estimate *in vivo* drug permeability due to their morphological and functional similarities. The P_gp_ inhibitor parameter of 2d is 0.014, which is in the range of 0−0.3, indicating **2d** as an inhibitor of P-glycoprotein. The P_gp_ substrate value of **2d** is 0.007 > 0, which shows that the modulation of P-glycoprotein-mediated transport has significant pharmacokinetic implications for P_gp_ substrates, which may either be exploited for specific therapeutic advantages or result in contraindications. The human intestinal absorption (HIA) value of **2d** is 0.008 > 0, which indicates that the close relationship between oral bioavailability and intestinal absorption has also been proven, and HIA can be seen as an alternative indicator for oral bioavailability to some extent. As shown in the electronic supplementary material, table S5, the properties of the drug distribution of the compound are in optimal ranges. The plasma protein binding (PPB) value of compound is 99.73% > 90%, which indicates that one of the major mechanisms of drug uptake and distribution is through PPB; thus, the binding of a drug to proteins in plasma has a strong influence on its pharmacodynamic behaviour. PPB can directly influence the oral bioavailability because the free concentration of the drug is at stake when a drug binds to serum proteins in this process. The (blood–brain barrier) penetration value of the compound is 0.027 > 0, thus the compound has moderate penetration via PPB. The Fu value is 0.929%, which is lower than that of the standard value, which also indicates that the compound has low fraction unbound in plasma. Most drugs in plasma will exist in equilibrium between either an unbound state or bound to serum proteins; in this case, they can traverse cellular membranes or diffuse. As indicated in figure 7, the properties of the drug metabolism of the compound are within the permissible ranges. Based on the chemical nature of biotransformation, the process of drug metabolism reactions can be divided into two broad categories: phase I (oxidative reactions) and phase II (conjugative reactions). The human cytochrome P450 family (phase I enzymes) contains 57 isozymes, and these isozymes metabolize approximately two-thirds of known drugs in humans, with 80% of this attributed to five isozymes—1A2, 3A4, 2C9, 2C19 and 2D6. Most of these CYPs responsible for phase I reactions are concentrated in the liver. The compound showed strong inhibition against human cytochrome P450 (CYP) enzymes, including CYP1A2, CYP2C19, CYP2C9, CYP2D6 and CYP3A4. It also demonstrated substrate properties for these enzymes, as presented in the electronic supplementary material, table S6. The properties of the drug excretion of the compound, such as CL and T1/2, are presented in the electronic supplementary material, table S7. The value of CL is 4.386, which is lower than 5−15 ml min^−1^ kg^−1^. This proved that the clearance of a drug is low, and clearance is a significant pharmacokinetic parameter that defines, together with the volume of distribution, the half-life and thus the frequency of dosing of a drug. The properties of the drug toxicity of the compound are given in the electronic supplementary material, table S8. Human hepatotoxicity and drug-induced liver injury have very high rates, which proves compound-induced liver injury is of great concern for patient safety and a major cause for drug withdrawal from the market. Others are in optimal ranges. As indicated in the electronic supplementary material, table S9, the compound exhibits acceptable environmental toxicity properties. As demonstrated in the electronic supplementary material, table S10, the toxicophoric rules of the compound are very good.

## 4. Conclusion

Novel coumarin derivatives (**2a**−**2j**) were successfully synthesized via the Mannich reaction. The compound **2d** showed strong anti-cancer activity against MCF-7 (IC_50_ = 2.54 ± 0.12 µM), surpassing camptothecin (IC_50_ = 3.76 ± 0.15 µM). Compounds **2a** and **2f** exhibited notable antibacterial effects against *B. subtilis*, while compound **2i** showed significant antioxidant activity (SC_50_ = 7.36 ± 0.18 µM). MD simulations confirmed the stable interaction of compound **2d** with 1T8I, with key amino acid interactions persisting up to 90 ns. Cytotoxicity assays on LLCPK1 cells (IC_50_ = 94.6−97.26 µM) indicated good selectivity, supporting their therapeutic potential. Further studies should focus on *in vivo* validation, molecular mechanism exploration, structural optimization and expanded antimicrobial screening to enhance the applicability of these compounds.

## Data Availability

The data supporting this article are reported in the supplementary material [57]. The authors have used software, such as Chemdraw Ultra-12.0.2. 1076 @ 1986-2010 CambridgeSoft; Avogadro software: version 1.1.1; Discovery Studio Visualizer v. 21.1.0.20298, copyright, Dassault System Biova Corp; Molegro Molecular Viewer 2.5: MMV 2012.2.5.0-2012-10-10, CLC bio company; PyMOL software: version 2.5.4 Copyright Schrodinger, LLC; AutoDockTools-1.5.6rc3, Desmond Schrodinger 2018 version 4: D. E. Shaw Research: Desmond Registration, to calculate the simulation model and web platform for the calculation of pharmacokinetic-ADMET model.
